# Quantifying burden of disease to support public health policy in Belgium: opportunities and constraints

**DOI:** 10.1186/1471-2458-14-1196

**Published:** 2014-11-21

**Authors:** Brecht Devleesschauwer, Charline Maertens de Noordhout, G Suzanne A Smit, Luc Duchateau, Pierre Dorny, Claudia Stein, Herman Van Oyen, Niko Speybroeck

**Affiliations:** Institute of Health and Society (IRSS), Faculty of Public Health, Université catholique de Louvain, Brussels, Belgium; Department of Virology, Parasitology and Immunology, Faculty of Veterinary Medicine, Ghent University, Salisburylaan 133, 9820 Merelbeke, Belgium; Department of Comparative Physiology and Biometrics, Faculty of Veterinary Medicine, Ghent University, Merelbeke, Belgium; Department of Biomedical Sciences, Institute of Tropical Medicine, Antwerp, Belgium; Division of Information, Evidence, Research, and Innovation, World Health Organization Regional Office for Europe, Copenhagen, Denmark; Department of Public Health and Surveillance, Scientific Institute of Public Health, Brussels, Belgium

**Keywords:** Belgium, Disease burden, Disability-adjusted life years, Health policy

## Abstract

**Background:**

To support public health policy, information on the burden of disease is essential. In recent years, the Disability-Adjusted Life Year (DALY) has emerged as the most important summary measure of public health. DALYs quantify the number of healthy life years lost due to morbidity and mortality, and thereby facilitate the comparison of the relative impact of diseases and risk factors and the monitoring of public health over time.

**Discussion:**

Evidence on the disease burden in Belgium, expressed as DALYs, is available from international and national efforts. Non-communicable diseases and injuries dominate the overall disease burden, while dietary risks, tobacco smoking, and high body-mass index are the major risk factors for ill health. Notwithstanding these efforts, if DALYs were to be used for guiding health policy, a more systematic approach is required. By integrating DALYs in the current data generating systems, comparable estimates, rooted in recent local data, can be produced. This might however be hampered by several restrictions, such as limited harmonization, timeliness, inclusiveness and accessibility of current databases.

**Summary:**

Routine quantification of disease burden in terms of DALYs would provide a significant added value to evidence-based public health policy in Belgium, although some hurdles need to be cleared.

## Background

The main goal of public health policy is to promote, enhance and protect the population’s health. This requires information on the health status of the population, often referred to as the “burden of disease”. More than just the presence/absence of specific diseases and conditions, disease burden encompasses a comprehensive quantification of the physical and psychosocial health impact of diseases, conditions, and risk factors [1].

Evidence on the disease burden is important for decision-making processes within the health sector. In order to make relevant decisions and set appropriate priorities, policy makers need to be informed about the size of health problems in the population, the groups that are particularly at risk, and the trends in the state of health over time. In addition, an accurate estimate of the population’s health status can be used for determining the expected health care use and is vital for prioritizing effective interventions and evaluating their impact and cost-effectiveness (e.g., by integrating them in generalized cost-effectiveness analyses [2]).

The disease burden of the population can be described by a variety of indicators. Indeed, public health is a multifactorial phenomenon with many facets and different ways to measure it. Typical indicators of population health are life expectancy, cause-specific mortality rates, numbers of new and existing cases of specific diseases (i.e., incidence and prevalence), perceived health, the occurrence of physical and mental limitations and disability, but also more indirect measures, such as absenteeism, incapacity of work, and the use of medical facilities and the associated costs. However, all these indicators highlight only one facet of public health, i.e., either mortality or morbidity.

Summarizing public health in terms of mortality-based indicators, such as life expectancy, dates from the time when only reliable data for mortality existed. In many countries, however, one has been confronted with an epidemiological transition of public health problems. The importance of early mortality due to plagues and famines has been replaced by chronic, non-communicable diseases, while communicable diseases remain a real threat, causing a “double burden” [3]. Cardiovascular diseases and cancers have replaced infectious diseases as the main causes of death. However, these diseases are also associated with an important morbidity component, due to the life prolonging effect of continuously improving medical practice [4]. Moreover, not only an extended life expectancy per se is aimed for, living these extra years in good health has become just as important [5]. As a result, current health policy requires a global overview of public health, one that combines morbidity and mortality and takes account of health-related quality of life (HRQoL; [6]).

Given the importance of combining morbidity and mortality, the last few decades have seen important methodological advances in so-called summary measures of population health (SMPH; [7]). By and large, SMPHs may be divided into two broad families, namely health expectancies and health gaps. Metrics of each family combine morbidity and mortality into a single figure. Health expectancy-based metrics, such as Disability-Free Life Expectancy (DFLE), Healthy Life Years (HLY), and Disability-Adjusted Life Expectancy (DALE), translate these indicators into a health-adjusted life expectancy; health gap metrics, such as the Disability-Adjusted Life Year (DALY), translate these indicators into a number of life years lost due to bad health and mortality.

Driven by the influential *Global Burden of Disease* (GBD) projects initiated in the early 1990s, the DALY has become the dominant SMPH [8–12]. In the remainder of this debate, we will therefore outline the composition of the DALY metric and its data needs. Next, we will summarize existing DALY estimates for Belgium, providing an overview of the current state-of-knowledge on the burden of disease in Belgium. We will conclude by discussing the added value and potential hurdles of routine DALY calculation for Belgian public health policy.

## Discussion

### Disability-adjusted life years

#### What are DALYs?

The DALY concept was developed for the World Bank’s *World Health Report 1993, Investing in Health* [13]. To estimate the global burden of disease, the World Bank required a metric that allowed comparing health across countries. Equity issues were therefore central concepts in the development of DALYs [14, 15].

DALYs measure the health gap from a life lived in perfect health, and quantify this health gap as the number of potentially healthy life years lost due to morbidity, disability and mortality. A disease burden of 100 DALYs per 1000 people-year would thus imply a loss of 100 healthy life years per 1000 people per year. Diseases or risk factors accounting for more DALYs thus have a higher public health impact.

The DALY is composed of a morbidity and a mortality component [14–16]. Morbidity is quantified in terms of *Years Lived with Disability* (YLDs), the loss of healthy life years from living in a less-than-perfect health state. YLDs for a given health state are calculated by multiplying the number of incident cases with the duration and the disability weight of the health state. An alternative, prevalence-based version of the YLD, introduced in the GDB 2010 study, is defined as the number of prevalent cases multiplied with the disability weight [1]. In both versions, the disability weight is a crucial component, reflecting the relative reduction in HRQoL on a scale from zero (perfect health) to one (worst possible health state). Mortality is quantified in terms of *Years of Life Lost* (YLLs), the loss of healthy life years from dying before a predefined life expectancy. YLLs are calculated by multiplying the number of deaths with the residual life expectancy at the age of death. The standard DALY formulas may be extended by applying age weighting or time discounting [14–16]. These so-called social weighting functions are however not accepted by all authors [17, 18]. As a result, the use of age weighting and time discounting is declining, even in the most recent updates of the GBD [1, 12].

#### Data needs

It is obvious that the quality of the DALY estimate directly depends on the quality of the input data [19]. To obtain the most accurate DALY estimate, it is therefore necessary to have reliable incidence (or prevalence) and mortality data. Ideally, one would like to have data from a nationally representative system that continuously monitors the occurrence of all disorders in the population, based on a set of clearly defined diagnostic criteria. As such registration does not exist, one must resort to what exists. In Belgium, several large and important data generating systems are in place:

–The national health surveys (performed in 1997, 2001, 2004, 2008 and 2013; https://his.wiv-isp.be/)
–The national cancer registry (http://www.kankerregister.org/)
–The 40 national reference centers and 16 national reference laboratories for human microbiology (https://nrchm.wiv-isp.be/; [20])
–The network of sentinel GPs and sentinel microbiology laboratories (https://www.wiv-isp.be/epidemio/epien/index8.htm; [21])
–The mandatory reporting of specific agents.

Taking into account the level of under-reporting and under-ascertainment, these data sources can provide valuable information on the incidences of the included diseases and risk factors.

The direct use of healthcare providers’ data is difficult in Belgium, given the stringent privacy rules in place [22]. However, certain data are made available as *Minimal Clinical*, *Hospital*, *Nursing*, and *Psychiatric Data*. These data sources are merely focused on the financial impact of diseases, and thus provide valuable information for estimating the direct health-related costs [23]. The announced intention to update the disease classification system to ICD-10-BE (*International Classification of Disease, 10*^*th*^*Revision – Belgian Modification*) in the domain *Medical Data* of the *Minimal Hospital Data* [24], might prove valuable for health impact assessment studies, given that non-aggregated data are made available.

In addition to the national data collection systems, there are various data collection mechanisms at the regional level. However, the routine use of these data sources to generate nationally relevant burden estimates might be problematic, due to the lack of harmonization between databases and data collection procedures in the different Belgian regions. Moreover, the diseases that are being registered are different between regions, making the availability of the routine data incomplete for a part of the country.

When local data are not readily available, one may resort to international databases or data from neighboring countries. However, Vanthomme *et al.* [25] report that lack of timeliness can be an important constraint to the use of three major health databases (i.e., WHO-HFA, OECD, and EUROSTAT). Also, when extrapolating data from neighboring countries, one assumes that the Belgian health situation is similar to that of its neighbors.

The lack of data harmonization in the Belgian system is mainly because of the absence of a unique national system of health data collection. There are several individual initiatives from public and private institutions, but communication between these different actors is poor. Several registries for the same disease exist (e.g. diabetes registry), but the stage from which the patient is included into a certain registry is sometimes poorly defined. Moreover, the codes used to define the disease, the identification of the patients and the follow-up period are different between registries making them impossible to merge. The split of health competences between federal state, regions and communities is one of the explanations of this disparate system of health data collection.

Finally, several diseases are not well registered in Belgium, such as foodborne diseases (FBD) and especially diseases caused by chemicals. The impact of FBD on public health is difficult to evaluate and these diseases are not prioritized in Belgium, creating a vicious circle of lacking data [26].

### The burden of disease in Belgium

Estimates on the burden of disease in Belgium are available from both international and national efforts. However, if disease burden were to support policy, a more systematic approach is required, generating comparable estimates rooted in recent, local data.

#### International efforts

To date, the two most comprehensive sources of disease burden estimates for Belgium are the most recent GBD studies conducted by the World Health Organization (WHO) and the Institute for Health Metrics and Evaluation (IHME). The results of WHO’s so-called Global Health Estimates (GHE) are available online as a series of spread sheet documents (http://www.who.int/healthinfo/global_burden_disease/estimates/en/index2.html). The results of IHME’s GBD 2010 study are available online via interactive data visualization tools (http://www.healthdata.org/gbd/data-visualizations), via detailed databases [27] and via a summary report [28, 29].

According to WHO GHE, the total disease burden in Belgium in 2012 was 29.468 DALYs per 100,000, of which 41% was due to morbidity and 59% due to mortality. Non-communicable diseases attributed 85%, injuries 10%, and the group of communicable, maternal, perinatal and nutritional conditions the remaining 5%. The group of non-communicable diseases was dominated by malignant neoplasms and cardiovascular diseases (each contributing ~20%), followed by mental, neurological and musculoskeletal disorders (each contributing ~10%). Falls, road traffic accidents and self-harm were each responsible for 20-30% of the entire injury burden. Lower respiratory infections dominated the communicable disease burden (37% of communicable, maternal, perinatal and nutritional conditions). Table 1 shows the top 20 causes of DALYs, YLDs and YLLs for Belgium in 2012 according to WHO, and their change in ranking since 2000. There has been a significant increase in the importance of Alzheimer’s disease and falls, while the relative importance of road traffic accidents and breast cancer has decreased.

**Table 1:** **Top 20 causes of Disability-Adjusted Life Years (DALY), Years Lived with Disability (YLD) and Years of Life Lost (YLL) in Belgium, 2012, according to WHO global health estimates**

Rank	DALY		YLD		YLL	
1	Ischaemic heart disease	[=]	Back and neck pain	[=]	Ischaemic heart disease	[=]
2	Other circulatory diseases	[=]	Unipolar depressive disorders	[=]	Trachea, bronchus, lung cancers	[=]
3	Trachea, bronchus, lung cancers	[=]	Falls	[=]	Other circulatory diseases	[=]
4	Back and neck pain	[+1]	Other musculoskeletal disorders	[=]	Stroke	[=]
5	Alzheimer’s disease and other dementias	[+6]	Alcohol use disorders	[=]	Other malignant neoplasms	[+1]
6	Chronic obstructive pulmonary disease	[=]	Alzheimer’s disease and other dementias	[=]	Chronic obstructive pulmonary disease	[+1]
7	Unipolar depressive disorders	[=]	Chronic obstructive pulmonary disease	[=]	Alzheimer’s disease and other dementias	[+6]
8	Stroke	[−4]	Ischaemic heart disease	[=]	Self-harm	[−3]
9	Falls	[+3]	Anxiety disorders	[=]	Colon and rectum cancers	[=]
10	Other malignant neoplasms	[−1]	Migraine	[=]	Lower respiratory infections	[+1]
11	Alcohol use disorders	[+2]	Osteoarthritis	[=]	Breast cancer	[−1]
12	Self-harm	[−2]	Road injury	[=]	Other digestive diseases	[=]
13	Other musculoskeletal disorders	[+2]	Asthma	[=]	Road injury	[−5]
14	Colon and rectum cancers	[+2]	Schizophrenia	[=]	Cirrhosis of the liver	[=]
15	Road injury	[−7]	Other circulatory diseases	[+1]	Other unintentional injuries	[+4]
16	Other digestive diseases	[+2]	Drug use disorders	[−1]	Pancreas cancer	[+1]
17	Breast cancer	[−3]	Bipolar disorder	[=]	Other respiratory diseases	[−2]
18	Lower respiratory infections	[−1]	Other unintentional injuries	[=]	Other infectious diseases	[+2]
19	Other unintentional injuries	[+1]	Rheumatoid arthritis	[=]	Lymphomas, multiple myeloma	[−1]
20	Anxiety disorders	[+2]	Stroke	[=]	Falls	[+3]

The change in rank since 2000 is given between brackets, with ‘+’ indicating an increase in rank, ‘–’ a decrease in rank, and ‘=’ a status quo.

The GBD 2010 study showed a broadly similar picture [28, 29], which is not surprising as WHO GHE adopted most GBD 2010 YLD estimates and used the same mortality data for calculating YLLs. Nevertheless, there are slight differences in methods, data sources and groupings of causes [12]. Table 2 shows the top 20 causes of DALYs, YLDs and YLLs for Belgium in 2010 according to IHME, and their change in ranking since 1990. In this 20-year period, the most striking increases are again those of the neurological, mental and musculoskeletal disorders.

**Table 2:** **Top 20 causes of Disability-Adjusted Life Years (DALY), Years Lived with Disability (YLD) and Years of Life Lost (YLL) in Belgium, 2010, according to IHME GBD 2010**

Rank	DALY		YLD		YLL	
1	Ischaemic heart disease	[=]	Low back pain	[=]	Ischaemic heart disease	[=]
2	Low back pain	[=]	Major depressive disorder	[=]	Lung cancer	[+1]
3	Stroke	[=]	Falls	[+2]	Stroke	[−1]
4	Lung cancer	[=]	Neck pain	[−1]	Self-harm	[+1]
5	COPD	[=]	Other musculoskeletal	[−1]	COPD	[+1]
6	Falls	[+3]	COPD	[=]	Colorectal cancer	[+1]
7	Major depressive disorder	[=]	Alzheimer’s disease	[+5]	Road injury	[−3]
8	Alzheimer’s disease	[+8]	Diabetes	[+2]	Lower respiratory infections	[+2]
9	Self-harm	[−1]	Ischaemic heart disease	[=]	Breast cancer	[−1]
10	Road injury	[−4]	Migraine	[−2]	Alzheimer’s disease	[+6]
11	Neck pain	[−1]	Anxiety disorders	[−4]	Other cardio & circulatory	[−2]
12	Other musculoskeletal	[+2]	Drug use disorders	[−1]	Cirrhosis	[−1]
13	Diabetes	[−2]	Osteoarthritis	[+1]	Pancreatic cancer	[+4]
14	Colorectal cancer	[−1]	Road injury	[−1]	Diabetes	[−1]
15	Other cardio & circulatory	[=]	Alcohol use disorders	[=]	Falls	[+5]
16	Breast cancer	[−4]	Asthma	[+1]	Prostate cancer	[+3]
17	Lower respiratory infections	[=]	Schizophrenia	[+2]	Brain cancer	[+1]
18	Migraine	[+1]	Other hearing loss	[−2]	Leukaemia	[+3]
19	Anxiety disorders	[+1]	Dysthymia	[+2]	Stomach cancer	[−7]
20	Cirrhosis	[−2]	Bipolar disorder	[=]	Chronic kidney disease	[+2]

The change in rank since 1990 is given between brackets, with ‘+’ indicating an increase in rank, ‘–’ a decrease in rank, and ‘=’ a status quo.

GBD 2010 further provides estimates on the contribution of risk factors to the disease burden. In the general population, the three most important risk factors were dietary risks (e.g., high sodium, low fruits and vegetables), tobacco smoking, and high body-mass index. Second-hand smoke exposure was a leading risk factor for children under 5, while alcohol use was the major risk factor for adults aged 15–49.

**Figure 1:**
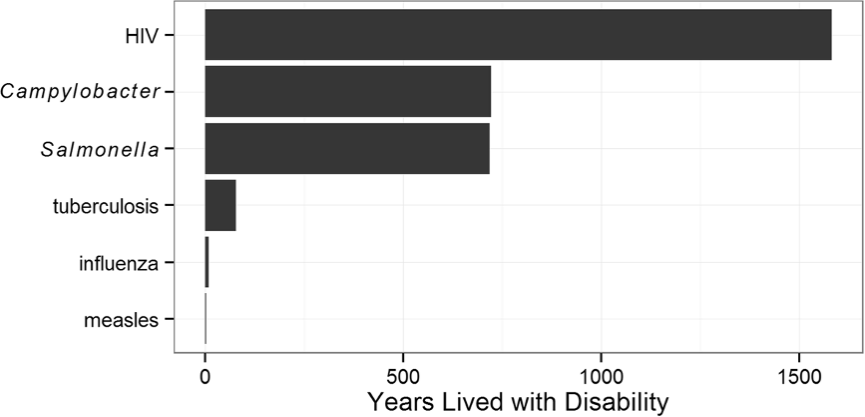
**Years lived with disability calculated in a pilot study on communicable diseases in Europe, based on data from 2003 to 2005** [30, 31] **.**

In addition to these global consortia, the European Centre for Disease Control (ECDC) is currently undertaking a burden of communicable disease study in Europe [30]. In a pilot study, the burden of six infectious diseases was quantified, though only YLDs could be calculated (Figure 1; [31]).

#### National efforts

So far, only few national efforts have been undertaken to study the disease burden in Belgium. The DALY as policy-relevant measure for Belgium was first described by Baert *et al.* [32], in the Flemish *Health Indicator Report 1998*. To demonstrate the use of DALYs, the authors initiated a pilot study in which they quantified the Flemish disease burden for reference year 1997 [33]. The list of included diseases and risk factors was inspired by the Dutch national disease burden study [34]. DALYs were calculated based on the Flemish life expectancy table, using non-uniform age weighting and a 3% time discount rate, making these estimates incomparable with the aforementioned GBD estimates.

**Figure 2:**
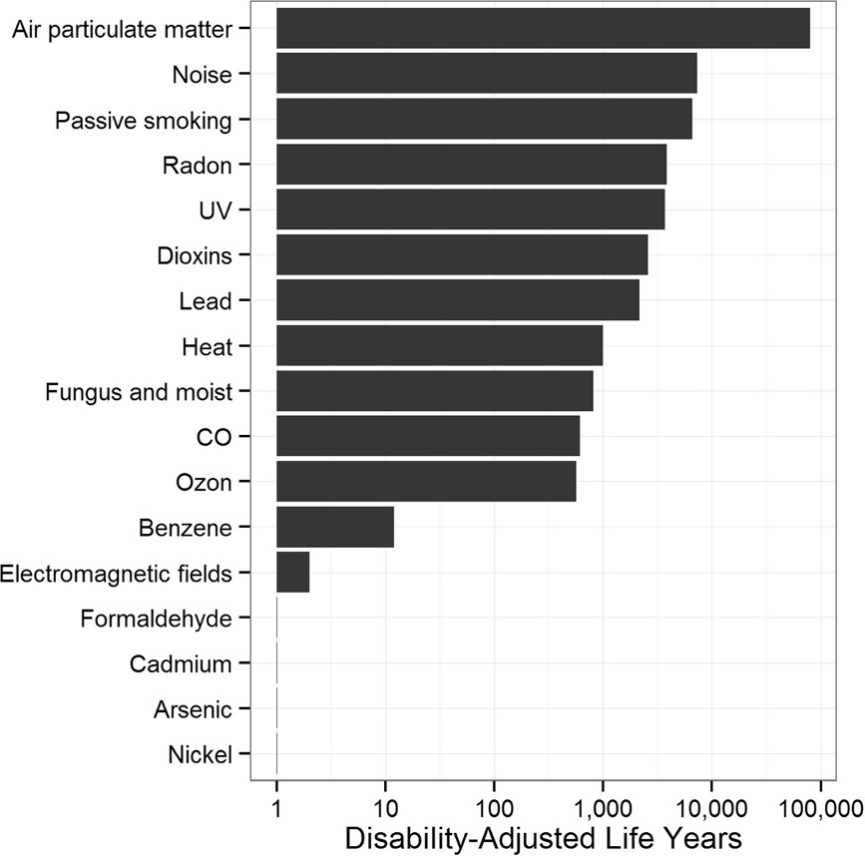
**Disability-adjusted life years for environmental risk factors in Flanders, based on data from 1998 to 2011** [35]**.** Note that the x-axis uses a logarithmic scale.

More recently, the Flemish Institute for Technology and Development (VITO) assessed the burden of environmental risk factors in Flanders, commissioned by the Flemish Environment Agency (VMM; [35]). Figure 2 summarizes their findings, showing air particulate matter to be the most important environmental risk factor.

In addition to these larger studies, several researchers estimated the burden of specific health conditions in Belgium. However, as DALY calculation methods differed across studies, care should be taken in comparing the resulting estimates. Stassen *et al.* [36] estimated the mean burden due to transportation noise in Flanders in 2004 to be 20,517 DALYs, or 342 DALYs per 100,000. Dhondt *et al.* [37] quantified the burden of road traffic accidents in Flanders and Brussels. They estimated a total of 182,379 DALYs, or 1030 DALYs per 100,000. In absolute numbers, car users contributed most DALYs, whereas per travelled km, motorcyclists contributed most. Henrard *et al.* [38] estimated the burden of haemophilia in Belgium in 2011 at 145 DALYs (95% credible interval [CI]: 90–222), or 1.3 DALYs per 100,000 (95% CI: 0.8–2.0).

## Summary

By quantifying the total disease burden and the contribution of different diseases and risk factors, DALYs are a highly valuable measure to set priorities for public health research and policy. Furthermore, if data allow, DALYs may be calculated for different socioeconomic groups or geographic regions, allowing for a more detailed perspective on public health. By regularly updating the DALY estimates based on the best available data, trends in public health may be monitored over time, and the impact of macro-level policies may be evaluated. As a result, DALYs may be important tools to support policy that aims to improve general population health and reduce health inequalities [39]. For this reason, the IHME is initiating national and subnational burden of disease studies, worldwide [40]. The WHO Regional Office for Europe is collaborating with IHME to facilitate national burden of disease study in the European region and enhance consistency of burden estimates.

Current DALY estimates for Belgium highlight the importance of non-communicable diseases and injuries. However, several constraints can be identified that might hamper the policy relevance of the currently available estimates. First, most DALY estimations remained academic exercises, with little or no direct knowledge transfer to the concerned policy instances. Indirect knowledge transfer may have occurred by referring to existing burden estimates in research proposals, but the effect is difficult to assess. Second, while global estimates provide a broad overview of the health status in Belgium, it remains a question to what extent these estimates are grounded in the best available local data. There may also be issues related to timeliness and ownership of these global estimates. Third, while national research groups did more efforts to apply local data sources, there appears to be little consistency in the applied DALY calculation methodology. As a result, the individual DALY studies cannot be combined to obtain a comparable evaluation of Belgians’ health. Researchers are therefore advised to calculate DALYs under different social weighting scenarios, and to present at least relative DALY estimates (e.g., DALYs per 100,000 people-year).

To overcome these limitations and generate a systematic and truly comparable measurement of Belgians’ health, DALYs should be integrated in the different official data collection systems. This is already the case in the Netherlands and Australia, where DALYs are guiding health policy since the past 10–20 years [34, 41]. In Belgium, various large and important data generation systems are in place that could provide the data required for calculating DALYs. However, there are also some potential hurdles, such as a lack of timeliness of certain databases, a restricted access to hospital data for routine use, a limited harmonization between regional databases, and the absence of certain diseases from the major databases. Given the increasing importance of neurological, mental and musculoskeletal disorders, the absence of comprehensive, harmonized databases for these disorders is particularly striking. Nevertheless, we believe that the routine quantification of disease burden in terms of DALYs would provide a significant added value to public health policy in Belgium and should be integrated in all national mechanisms for the translation of evidence into policy.

## Abbreviations

DALE: Disability-adjusted life expectancy
DALY: Disability-adjusted life year
DFLE: Disability-free life expectancy
DW: Disability weight
ECDC: European centre for disease control
FBD: Foodborne disease
GBD: Global burden of disease
HLY: Healthy life year
HRQoL: Health-related quality of life
IHME: Institute for Health Metrics and Evaluation
SMPH: Summary measure of public health
VITO: Flemish Institute for Technology and Development
VMM: Flemish Environment Agency
WHO: World Health Organization
YLD: Year lived with disability
YLL: Year of life lost.
